# On the Use of Mercury in the Venereal Disease

**Published:** 1837-10-01

**Authors:** 


### ON THE USE OF MERCURY IN THE VENEREAL DISEASE.

I. Practical Observations on the Venereal Disease and on the Use of Mercury. By *Abraham Colles*, M.D. One of the Surgeons of Doctor Stevens's Hospital, and lately Professor of Surgery in the Royal College of Surgeons, in Ireland. 8vo. pp. 351, Sherwood and Co. London, 1837.

II. The Works of John Hunter, F.R.S. With Notes. Edited by *James F. Palmer*, Senior Surgeon to the St. George's and St. James's Dispensary, &c. &c. In Four Volumes. Illustrated by a Volume of Plates in Quarto. Vol. II. London, 1835.

III. Historical and Medical Researches into the Origin, Nature, and Treatment of Syphilis. By *M. Devergie*, Sen. Translated by *John Sinclair Innes*, Surgeon. 18mo. pp. 63, 1837.

The modern Lacon, in one of his aphorisms, declares that all which medicine has been able to effect in sixty centuries, has been the discovery of two specifics, mercury and brimstone. If this had been all, it would have been but little, nay less than even the sarcastic epigrammatist supposed, for the first specific, mercury is considered by many to be no specific at all. A pregnant instance of this curious fact may be found in the juxta position of the three works, whose titles we have placed at the commencement of this article.

The first, by Dr. Colles, is devoted to the object of shewing the necessity of salivation for syphilis: while the third, by M. Devergie, is intended to point out the folly, obstinacy, and ignorance of those who pursue such irrational treatment.

The enthusiasm of our neighbours appears strange, almost ridiculous, in the eyes of a people so practical and sober as ourselves. We cannot repress a smile when we see such enthusiasm excited by occasions which seem to be little calculated for the purpose, and that smile is too apt to be tinctured with contempt, when substantial advantages and positive results are sacrificed for that honour or glory, which are ever in a Frenchman's heart and mouth. Yet our more phlegmatic temperaments, so useful to us as a commercial people, are perhaps too little qualified by a generous desire of mere distinction, and the constitution of society, which grows out of our national character, has contributed to give a mercenary tone to every calculation. Where a French savant aspires to the cross of the legion of honour, an English philosopher is pondering on what may make his fortune: and while the science of the former is devoted to the improvement of the arts of life, that of the latter is more profitably engaged in their application. But to return to our proper subject.

Dr. Colles draws the following picture of the present state of our knowledge with respect to syphilis:—

"When we recollect, that for three centuries and upwards, medical men throughout all Europe, have been employed most extensively and uninterruptedly in the treatment of this disease; that during almost the entire of that period a remedy has been known which possessed the power of curing this disease; that this remedy, since its first discovery, has undergone a variety of modifications and combinations, supposed to render it more suited to particular states or forms of the disease; that, in addition to this, some other remedies, and other plans of treatment, have been found to effect its cure: when, I say, we take all these facts into account, surely it might be reasonably expected, that with all these advantages, we should by this time, have arrived at a knowledge of fixed rules for its treatment, and have acquired such a command over it, as to be able to say, that we can control its course and check its ravages.

Yet I think it is not an exaggeration to assert, that at no period, since the disease was first subdued by mercury, has the opinion of Practitioners been more divided and unsettled, or their treatment more wavering and unsuccessful.

Anxious to have this reproach wiped away, not merely because it involved the character of the Profession, but rather because it too clearly showed that the human race was suffering severely from our imperfect knowledge of the treatment of this disease, I sought to discover the causes of our backwardness. After mature consideration, I was led to attribute it principally to two causes:—

*First.* The imperfect knowledge we possess of the natural course or natural history of the Venereal Disease.

*Second.* The very imperfect knowledge we possess of the means of directing the operation of mercury, so as to make it act in a salutary manner; and the equally imperfect knowledge we have of the earliest phœnomena which would indicate that it is beginning to act as a poison rather than as a remedy." vi.

These are valid reasons for any communications from a practical and observant man, upon this apparently perplexing disease. It is for the same reasons that we have on several occasions devoted many pages of this Journal to the subject; and, for aught that we can see, if such reasons are to sway us, we have to devote many more pages still. The plan pursued by Dr. Colles may be stated in his own words.

"I have stated in detail such observations as I have made as to the progress or natural history of the Venereal Disease; and I have offered some remarks on the mode of administering mercury, so as to induce the salutary action of this medicine. I have, also, attempted to point out a few early indications, which denote that its action will become poisonous if its use be persevered in; and I have detailed many of the symptoms of Syphilis, and stated my opinion as to their appropriate treatment." vii.

"From this statement then, the reader will not expect to find in the following pages a systematic treatise on the Venereal Disease; on the contrary, some symptoms have not even been mentioned, and others have been noticed only in a cursory manner. The remarks which I have offered are merely the result of my own observations and reflections. Of what value these may prove, I leave to my professional brethren to decide; only assuring them, that the facts are stated with the most scrupulous fidelity.

To the observations on the use of mercury in Syphilis I have added some observations on the use of this medicine in the treatment of diseases not venereal. In this second part of the work I have studiously avoided any mention of its use in the diseases in which its efficacy is generally known and long established; and I have designedly and strictly confined myself to those in which it has been only very rarely employed, but in which I have had the good fortune to have found it a most active medicine, and a most speedy and invaluable remedy." viii.

The work is divided into two Parts. The first treats of the Use of Mercury in Venereal Complaints—the second treats of the Use of Mercury in Affections of the Nervous System. The first part contains fifteen Chapters, with the following titles. 1. On the Natural History of the Venereal Disease; 2, On the Administration of Mercury; 3, On the Same; 4, On Chancre; 5, On Bubo; 6, On a Disease of the Lymphatic Glands of the Groin, attended with Peculiar Symptoms; 7, On Secondary Symptoms; 8, On Venereal Diseases of the Mouth, &c.; 8, (*bis*), On Venereal Eruptions, Nodes, and Diseases of the Testicle; 9, On the Use of Mercury in Venereal Hectic Fever; 10, (*bis*), On Mercury during Venereal Eruptions; 11, On the Treatment of Syphilis in Scrofulous Patients; 12, On Minute Doses of Mercury in Late and Chronic Bubo; 13, On Syphilis in Infants; 14, On Pseudo-Syphilis; 15, On the Non-Mercurial Treatment of Syphilis.

A glance at the headings of these Chapters is sufficient to demonstrate the absence of any natural arrangement of the subjects upon which the author treats. We may therefore take any liberty we please with such desultory observations, and throw together parts which are disjoined in the original.

The present article will be confined to the examination of the propriety of exhibiting mercury in syphilis, and to the natural course of the latter. We shall therefore begin with the last Chapter of Dr. Colles' book, and place by its side the observations of M. Devergie. We will commence with the latter, and we think it will soon appear that the extravagant enthusiasm of his nation is displayed in a striking manner in his pages.

It is unnecessary for us to enter on the history of the Venereal Disease, or of the application of mercury to its treatment. Suffice it to say, that the latter was not regulated by scientific principles, and that it was given to a ruinous extent as well as under most unfavourable circumstances. Various attempts have been made to substitute other remedies for mercury in the treatment of syphilis. Each has been partially, none wholly successful. As the horror of mercury has long struck deep root into the minds of men, these failures must be deemed presumptive evidence, that common experience has decided against the general success of anti-mercurial methods.

But although these did not supersede the use of mercury, they opened the eyes of men of observation to the errors which had prevailed with respect to the absolute necessity for the latter, and they tended strongly to correct the abuses which had prevailed in its administration. Salivation was carried to a less frightful extent, and it began to be a question what symptoms were, and what were not adapted for its application.

It was about the beginning of the present century, that the attempts to cure syphilis without the aid of mercury, were once more generally made, and that the numerical method was extensively employed as a test of their success. Those attempts were certainly commenced and carried on most fully by English, and, particularly, by our army surgeons, though M. Devergie has ingeniously endeavoured to arrogate the merit to his countrymen.

It would be saying no more than the truth, to observe, that the results took the Profession by surprise, and that numerical data, apparently unobjectionable, seemed to establish, in a decisive manner, the superiority of the non-mercurial over the mercurial method.

Leaving this Country for a season, we will shew from M. Devergie's pages, what has taken place in France. If his statements are to be implicitly received, the non-mercurial plan has flourished in that country, and the mercurial will, ere long, be almost extinct. After citing the names of a few who had distinguished themselves in opposing the old doctrines, M. Devergie goes on to observe :—

"The school not less celebrated for its physiological doctrines, (of which Val-de-Grâce has the honour not only of being the birth-place, but also of reckoning among its inhabitants the worthy founder of this school,) furnished physicians who, young in years, but strong in the good principles derived from so fruitful a source, entering the lists, contributed by their enlightened and novel researches to overthrow the ancient edifice, in order to elevate a new one more regular and more worthy of modern knowledge. Every day sees an increase in the partisans of this new treatment of syphilis. These ideas are not confined to the circle of French physicians; they have spread themselves among strangers —into England, America, Sweden, Denmark, and some parts of Germany, the same systems having been tried, the results have soon surpassed their expectations. We shall now be able to make known to you the results of this new method, and lay before you the observations collected as to it, as known under the name of the antiphlogistic system, known equally under the denomination of the simple and rational method; a method against which there has been an outcry from all parts, which has been condemned without being understood; been disputed every day, against which some men high in medical reputation, have daily hurled from the height of their chairs, anathemas and ridicule, and endeavoured to persuade their pupils that they are dragged into a false and deceitful route, in teaching them principles which they have it in their power to verify at the bedsides of the sick." 28.

Then comes the history of the progress of the anti-mercurialists in France.

"Chaussier, of immortal memory, regarded mercury as of little use in the treatment of syphilis. In 1811, the late Gerardot, the friend and rival of the author of 'the Chronic Phlegmasiæ,' taught me the art of curing all the chronic syphilitic phlegmasiæ by the dietary system; whilst at the same time, Baron Larrey brought about a happy change in the administration of mercury, that rendered the treatment more simple, easy and less dangerous, so that it has since been made the method of M. Pihorel. In 1811 and 1812, appeared successively the works of Caron upon a new mode of considering the syphilitic contagion; the considerations of M. Jourdan upon syphilis, the work of M. Keraudren, physician in chief to the navy, upon secondary syphilitic symptoms, and the exclusion of mercury from their treatment, and afterwards the valuable proposition of M. Broussais upon syphilis.

In 1824 were published the considerations of M. Lefévre upon the abuse of mercurials, and the essay upon the venereal disease of M. Dubled; the year 1826 saw appear the treatise on the venereal disease of M. Jourdan, the work upon the non-existence of the Syphilitic virus of Dr. Richmond, accompanied with tables of the first treatment without mercury, at the military hospital of Strasbourg; at the same period appeared the first part of the 'Clinique of Syphilis,' since become a complete treatise on Syphilis, with 125 plates, a work for which I had lately the respects of the academy. In 1827 was published in a medical journal, the report of the *College Sanitaire of Sweden*, upon the advantages of the treatment without mercury. Almost at the same period, also, the council of health of the army, thoroughly persuaded of the advantages to be obtained by changing the mercurial treatment, induced the heads of the military hospitals to make trial of the simple and rational treatment. In 1828 and 1829, M. Desruelles made known the statistical results of the simple treatment employed at Val-de-Grace for the cure of venereal diseases. I have published in, 1830, in the 15th, 16th, and 17th parts of my 'Clinique,' the tables of MM. Rapatel and Desruelles of Rennes, who in 1827-28-29, treated 1410 patients by the simple treatment. I must mention also the works of M. Willaume, surgeon-in-chief of the hospital of instruction at Metz, upon the advantages of the antiphlogistic system, and those of M. Puel of Marseilles. I have to tell that at Lille, Havre, and Bayonne, Drs. Delatour, Desjardins, and Becquart treated in the hospitals their patients by the simple treatment. I must not forget to mention the opinion of the late Delpech of Montpellier, who, imbued with ideas of virulence, treated, for a long time, the disease with mercury, lavished in all its forms, who, enlightened by degrees through experience, modified to such a state his ideas and treatment, that he published in 1828, that gonorrhœas and chancres might be completely cured by the efforts of nature solely, ninety to ninety-five times per cent., and that nature alone had cured one-half of the ulcers of the throat, pustules, radesyge, &c. Since 1830, I have received new proofs that, united with those communicated by my colleague and friend Dr. Desruelles, have gone to prove that at this time the simple rational antiphlogistic method ought to be adopted as a *general method* in the treatment of syphilis, whilst that the mercurial is used more as an *exceptional method*, employed in those cases where the simple system might have been found to be insufficient. Thus, at Rennes, the simple treatment, by procuring such advantages, the disproportion between the two kinds of treatment is become excessive. Out of 1505 patients affected with primary symptoms in 1827-28-29, 1187 had been treated without mercury, and 318 by mercury; whilst during eighteen months of 1830-31, 876 symptoms were cured by the simple method, and only forty-eight by the mercurial. This amelioration is still more sensible in the employment of the means for curing relapses; for in sixty-four relapses after one or other methods of treatment, twelve only were subdued by mercury; these twelve cases required for themselves alone 1082 days, whilst the fifty-two others took only 1863, so that it allowed to each case of the first eighty-one days, whilst for each of the second it was only thirty-five days. At Val-de-Grace Dr. Desruelles and myself have obtained summaries since 1825, with results not less advantageous. M. Barthélemy, at the military hospital of *la Maison-Blanche* (Paris), in 1833, had also to treat more than 700 syphilitic patients by the same method, and employed mercury only in a certain number of secondary affections. M. Kaisser, at the mililary hospital of Strasbourg, treated equally with the same success, by this method, for three consecutive years. At Algiers M. Fleschutt, surgeon-major to the hospital of the Dey, obtained also a great success by the simple and rational treatment, and for three years has to congratulate himself with having renounced mercurial preparation. At the syphilitic hospital of Paris, this method was made trial of by M. Cullerier; and the documents published by MM. Ruft and Pailloux of Chambereari, in 1831 and 1832, proving that the antiphlogistic treatment had been making progress for some years, and possesses real advantages, that are appreciated there. Indeed, out of 512 patients, 339 were cured by the simple treatment, the mean duration being thirty-two, whilst of the others it was forty-seven by the mercurial method. The relapses after the simple treatment have been one in twenty-eight; and the physicians of this establishment have been so sensible of the advantages of this simple and rational method, that there did not exist (in 1834) a more complete mercurial treatment, than that mercury is there employed as a powerful alterant of the economy only in those cases where the other methods have been used without success, and in general in small doses. For one year that I have had the charge of the first division of venereal patients at the military hospital of Gros Caillon, I have continued the same mode of treatment, and have not had recourse to mercury but in exceptional cases that have not yielded to a simple treatment." 33.

The following short notice of the labours of the English, and the candid exposition of our professional constitution are characteristic.

"The English physicians have entered the lists, and have treated without mercury, but not without exciting medicines, so that, with the exception of the works of Thompson, Turner, Brown, Ewans, Hennen, and some others, to which we may add the names of Guthrie and Sir Astley Cooper, we must reject the writings of the other English physicians, for they did not assist, but rather discredited this anti-mercurial system, without any advantage to their patients, who encumbered the hospitals uncured. But this need not appear astonishing to you, gentlemen, that it has been so long thus in a country where, with the exception of some of the principal physicians and surgeons, the physician and apothecary are one and the same; where the hand that writes the prescription at the bedside of the patient passes into the shop for its preparation." 34.

That "Old England" could be compared with "Young France" in intelligence, was, of course, not to be expected. The former has not enjoyed the advantage of a Val-de-Grace, of the great physiological doctrine, of the author of the Examen, nor of the sucking "physiologists," who have sprung from his scientific loins. But it is rather singular that, considering the unbounded love of M. Devergie for the anti-mercurialists and their views, he should have found it in his heart to hold at so cheap a price the labours of Thompson, Ferguson, Rose, Hennen, Guthrie, &c., and the reports of these Gentlemen extending from the year 1801 to 1823. According to his own shewing, it was only in 1811 that the late Gerardot taught him, M. Devergie, the art of curing the syphilitic phlegmasiæ dietetically, and it was only about the same time that the valuable propositions of M. Broussais appeared. Were the English, then, so very far behind? Has their application of statistics been so very late? With the utmost liberality, it is impossible not to perceive that M. Devergie is a vain-glorious man, puffed up with a frivolous amour-propre and amour de patrie.

But these are personal considerations, and, if we turn from them to the practical part of the question, we find that, in France, the non-mercurial treatment would appear to have met with a good deal of favour. M. Devergie introduces at the end of his work some results of trials conducted rather extensively by himself. But they are so confused that we must waive their insertion, and content ourselves with stating, that, like the reports from the non-mercurialists in this Country, they go to prove the superiority in point of success, of the simple antiphlogistic over the mercurial method.

So far as numerical comparisons go, the non-mercurial would appear to be superior to the mercurial mode of treatment. If any thing is clear this seems to be so. As this is an age in which a reverence for antiquity and a dislike of change are by no means prevalent faults, we might suppose that the mercurial treatment is pretty generally given up, and that the best informed surgeons have verified by their individual experience, results so decisive and so irrefragable as those established by the documents to which we have alluded.

We will turn to Dr. Colles and inquire of him the state of the case in Dublin.

"When this plan," (the non-mercurial treatment) "first attracted the notice of the surgeons of Great Britain, both my colleagues and myself," he says, "adopted it in our hospital. In general we confine this treatment to men who had not used any mercury; but, as in Dublin it is extremely difficult to meet with venereal cases in hospital, who have not applied to some apothecary, or received medicines at a dispensary, we could not adopt it as the general practice of the institution. However, we tried it until we all became convinced of this fact,—that it was not suited to patients who were obliged to earn their bread by labour; for we saw that after they had left the hospital, and got into employment, they generally found themselves weak, and unequal to their usual labour; and often, at the end of two or three months, they returned emaciated, pale, and enfeebled, in consequence of the hectical form of fever which was about to usher in a new series of venereal symptoms. Their stay in the hospital also proved, in general, very protracted; so that they then became impatient of this treatment, especially when they saw others with similar symptoms, in the same ward, have their complaints more quickly cured by the use of mercury. In private practice, also, I employed it for a time; but not finding it superior in point of quickness of cure, or of security against a relapse, and observing that these relapses were more frequently reiterated, in a short time I ceased to employ it, except at the express solicitation of the patient. But I had many opportunities of witnessing the results of the practice of some of my brethren, who adopted it more fully. Of course I could not know much of those cases in which this practice was successful; but in many of those who suffered from secondary symptoms, and from relapses of the different series of secondary symptoms, I had melancholy proof that this treatment was too often unequal to remove syphilis. No doubt fewer of the non-mercurial patients complained of affections of the bones, than those who had been ineffectually treated by mercury; but I saw instances of closed pupil and opaque lens, produced by iritis which had been neglected, not having been considered as venereal symptoms. I have seen many cases where the soft parts of the throat had suffered severe mutilations; and, above all, I had too many opportunities of watching the very slow and silent, but sure inroads, which the often-repeated attacks of secondary symptoms made on the constitutions of the patients; of witnessing this phenomenon,—that the venereal disease, from year to year, showed itself with less striking characteristics, while other diseases appeared to spring up; so that, for some months before the death of such patients, it would require a close examination to discover the one or two slightly-marked symptoms of syphilis which remained; and also required close research to trace the symptoms of apparently the last and fatal disease to its true source, the infection of syphilis. But on tracing the state of health, from the primary ulcer down to the final and fatal disease, I could clearly see that at no period was the unhappy sufferer altogether free from the venereal disease; so that both the patients and their friends, in many instances, lost sight of the original syphilitic disease, and referred the death to some other apparent cause, such as ascites, or some disease of the lungs. Nor is it to be wondered at, that non-professional persons should form such an erroneous opinion, seeing that the course of some of these fatal cases had occupied a period of four or five years, between the appearance of the primary ulcer and the fatal event. In a word, after the experience of one year's full trial of the non-mercurial plan, we have since, in our hospital practice, only employed it rarely, and generally in very mild slight cases of primary symptoms." 319.

After some other observations to which we shall presently advert, Mr. Colles goes on to observe:—

"I think it must, however, be admitted, that the non-mercurial plan has not fully answered the expectations of its early admirers. All will allow, that many cases of syphilis have resisted this treatment, and were afterwards cured by mercury. Let us revert to the history of its introduction. It was introduced at a time when (in Great Britain) mercury was rather in disrepute; and when in the Peninsular army the venereal disease, treated by mercury, was making frightful havoc among the soldiery. It was tried on a scale beyond that of any other medical experiment, and under circumstances the most favourable; the patients being subjected to military discipline and restraint. In civil life it would naturally obtain a preferrence from both patient and surgeon; the former would be relieved from all the horrors of a mercurial course, the latter find in it a line of practice simple, plain, and safe; one that required not any extensive observation of the venereal disease, or any nice and accurate judgment in the employment of the remedies. Yet it has not superseded the mercurial plan of treatment: on the contrary, it seems now, after a trial for twenty years, to have fewer and less warm admirers. This may possibly be fairly accounted for, by observing that this plan subjects the patient, especially when the symptoms are of an inflammatory character, to the most rigid quiet, even continued confinement to bed, to repeated bleedings both general and local, to the long-continued use of nauseating medicines, to frequent purging and very low diet. So that, during the treatment, the patient is subject to all this severe discipline; and, after it, suffers a proportional degree of weakness; and, to all this, I am disposed to add relapses more frequently repeated, although less severe than those which follow after the mercurial treatment." 320.

The preceding passages represent the results of the observations of practical men, not only in Dublin, but in London, and not only in these towns, but throughout the empire. How comes it that if the non-mercurial method were really the best, it has made no progress for many years, in that Country where practical medicine is most successfully cultivated? This is an argument which is not easily met. It is an argument not founded on partial, but on general statistics, not merely on a few experiments in which results may be vitiated by interest or by prejudice, but on the general experience of a profession.

If there were any substantial reasons for that profession shutting its eyes to the truth, if interest preponderated greatly on one side, such an argument would, of course, lose much of its force. But in a case where interest cannot warp the judgment, where the current of human motives and feelings runs rather in favour of the new doctrine than the old, it does appear monstrously incredible that error should be preferred to truth, and that men should obstinately adhere to a practice which every-day experience demonstrates to be unnecessary, unsuccessful, and cruel. Let those who will, adhere to so repugnant a belief; we cannot, and we do not.

Of this we are quite sure—that, amongst the hospital surgeons of London, who must necessarily have extensive opportunities of observation, there is hardly one who is now an anti-mercurialist. Mr. Liston is the only exception we are acquainted with, and Mr. Liston brought his views from Edinburgh. Mr. Liston's reputation, too, is rather that of the knife, than of ordinary practical attainments. But out of these same hospital surgeons we are acquainted with several, who were struck with the force of the facts detailed by the non-mercurialists, and who, in consequence, tried the antiphlogistic method. They have abandoned it because they found that on the whole it did not answer, and was inferior to the mercurial. In the Lock Hospital, of London, the former plan was adopted, and was given up for the same reason.

It is to be regretted that these experiments have not been conducted in so satisfactory a manner as they might have been. Surgeons having convinced themselves of the fallacy of the conclusions of the anti-mercurialists, have not taken the trouble to collect and to arrange the data which might numerically convince others. Hence the absence of those positive statements on the part of the mercurialists, which play so imposing and seductive a part in the pages of their adversaries. No doubt, there are some other reasons for the absence of statistical facts, but we fear that the principal is that apathy on scientific questions so conspicuous in this country, amongst all classes of medical men.

We believe, then, we are not far wrong in pronouncing that the common experience has decided against the non-mercurial method as one of general application. But we are sure that the same experience has decided in its favour as one of partial application. It would be the height of injustice to deny to the anti-mercurialists the merit (no slight one), of having mitigated the mercurial code—of having disabused the minds of the profession of the erroneous notions formerly so prevalent, with respect to the irremediable character of syphilitic symptoms by any but mercurial remedies—of having laid the first stone in the work of distinguishing the effects of the venereal poison from those of mercury, and the consequences of either, singly, from the joint operation of both. If the labours of these gentlemen have done this, which we are certain that they have, they have done much for science, and still more for humanity. We seldom see the mutilated countenances, the ulcerous skins, the crippled limbs which formerly constituted the too frequent disgrace of science and horror of society. The diseases of the bones, more particularly, have decreased, and the phagedænic ulcerations both primary and secondary, are comparatively rare, and, when present, are usually produced by obvious general causes. For all this, we repeat, that we must thank the anti-mercurialists.

But it is rather indirectly than directly that this good has been effected—it has not been by adopting the anti-mercurial, but by modifying the mercurial treatment. In this view, Dr. Colles coincides.

"We must acknowledge, "he says," that the profession is highly indebted to those who have lately introduced the non-mercurial plan of treatment; for we have now not only acquired a second line of treatment; for venereal cases, but, what is of the highest value, we have been released from an inveterate and deep-rooted error—from an unfounded conviction that the venereal disease could not be cured by the innate powers of the system, unless aided by mercury. I need not add, that all the opinions and practices consequent on this prejudice have been subverted." 319.

It is obvious, that the question of the superiority of one method of treatment to another, and particularly in such a case as the present, is not to be determined by argument but by fact. We have not therefore indulged in abstract or theoretical considerations, but simply balanced the evidence on one side against that of the other. The anti-mercurial evidence is very explicit, very direct, and, apparently, very strong—the mercurial is less direct, less explicit, and, consisting of the sum of general opinion, its cumulative strength is less capable of condensation and of demonstration. For our own parts, we must own that our experience has gone against the anti-mercurial method—that, so far as we have been able to judge, secondary symptoms have been frequent after its employment—that it is tedious at the best—uncertain in most cases—and unsuccessful in many. Like Dr. Colles, we have witnessed instances, where the patients have gradually declined and sunk from phthisis or from other visceral affections, and in which a candid review of the symptoms seemed obviously to refer them to syphilis, uncontrolled by mercury. In short, so far as our means of reasoning on facts will carry us, we are distinctly of opinion that a mild and a judicious employment of mercury, is the most successful mode of treating syphilis which has yet been discovered.

But perhaps there is no case in which more knowledge and more judgment is requisite, than in the application of mercury to venereal symptoms; and, perhaps, also, there is none, in which less knowledge and less judgment are too generally exhibited. Few, very few, either do or can study the venereal disease scientifically. The constitution of our hospitals, where alone medical men, young or old, can really become acquainted with it, seriously interferes with that study. Yet no man, from the chemist's apprentice upwards, seems to hesitate, for an instant, to treat a complaint which he must be conscious that he does not understand, or to exhibit a remedy which may do so much good or so much harm, just as it is appropriately administered or not. A singular and an unfortunate state of things.

Syphilis is not a simple series of phenomena, one of which has to the other a constant, and therefore a determinate relation; but a collection of symptoms which differ in appearance, in course, and in consequences. To increase the difficulty which must necessarily attend the study of such a disease, some of those symptoms are common to other conditions of system, besides the syphilitic. The phenomena of cachexia, for instance, are similar, whether that has resulted from the venereal poison, from mercury, from both conjoined, or from other causes. There is still another source of confusion. Syphilis is seldom allowed to run its course, unaltered by active treatment of some sort. From the operation of these circumstances, we are imperfectly acquainted with what Dr. Colles not inaptly terms the "natural history," of syphilis, and this imperfect acquaintance on the part of otherwise well-informed surgeons degenerates into very lamentable ignorance on that of too many others.

In order that mercury should be judiciously applied to the treatment of this complaint, the following facts must be determined:—1, the natural course and characters of syphilitic symptoms; 2, their modifications from the great constitutional diatheses, &c.; 3, the effects of mercury upon the system; 4, the effects of mercury upon the syphilitic symptoms, and the modifications of those effects by the disturbing circumstances of diet, season, and diathesis; 5, the effect of other remedies and modes of treatment; 6, the phenomena of those disorders, which, however induced, resemble syphilis, their discrimination, and their management.

It is almost evident, ex necessitate rei, that these circumstances should be determined before so grave a malady as syphilis *may* be, is treated by so powerful a remedy as mercury *is*. Yet how many practitioners, medical or surgical, have taken the trouble to resolve any one of them, and how few have hesitated to subject the disease in its worst and most complicated forms to a bold and hazardous empiricism? We leave the answer to our readers.

It might appear unnecessary to insist on a proper understanding of the modus operandi of mercury. Every student would almost feel affronted at the supposition that he did not know it thoroughly. Mercury, he would say, acts upon the liver, the skin, the salivary glands; and it exerts its specific action against syphilis. All this is very scholastic, but very superficial. If the same student were asked—"for what symptoms of syphilis is it useful, and for what injurious—how do you know when it is curing and when it is likely to aggravate the malady—what principles have you laid down for your guidance in its exhibition or its suspension?"—if asked we say such questions, most students and far too many men in practice would return but an unsatisfactory reply.

In an article on the venereal disease, published some four or five years ago, we remarked:—"we apprehend that the blind confidence in specifics is rapidly diminishing, and certainly the treatment of syphilis by its specific, mercury, is one of the most difficult problems in experimental medicine." To those sentiments we still adhere, but the case of mercury is a remarkable instance of the influence which a name exerts on the minds of the majority of men. The belief in its specific properties has been a damper on rational investigation, and mercury has for centuries been blindly administered, under circumstances the most opposite, and conditions the most unfavourable, simply because, being a specific, men would not give themselves the trouble of enquiring when its specific properties were best exerted, or how. The following observations by our author on this subject are so judicious that we quote them:—

"Hitherto, and indeed at this very day, surgeons have been very unreasonable in their expectations of the powers of mercury. No doubt many weak minds, misled by a name, have thought, if they administered mercury, which is considered a specific, they must cure the venereal disease. Yet it is strange that men have dealt more justly with other specifics: thus Peruvian bark is looked upon as a specific for the cure of intermittent fever; and it will, if judiciously administered, succeed in a great majority of cases of this disease; but still all must allow, that this specific may not only not cure, but may aggravate this disease, and even induce others of a more dangerous nature—if, for instance, it be given when the stomach and bowels are loaded; the same unfortunate result will also follow its use, if it be given at an improper period, ex. gr. during the paroxysm. Or, again, if it be directed in doses unfit in point of quantity, it must be acknowledged that it has failed; thus if given in very small doses, it will prove unequal to arrest the progress of the disease; or, if given in excessive doses, it will derange the stomach, so that this organ can no longer retain it. I need not add, that this specific must also fail if it be administered when the constitution has been much broken down, and when a material derangement of structure, in any important viscus, has been induced by the long continuance of the intermittent fever. Now when we come to recollect that the intermittent fever is a disease pursuing a single and regular course, we must see how much more easy it is to lay down plain and simple rules for its treatment, than we could do for a disease so complicated as syphilis is. Again, although Peruvian bark is in intermittent fever, and some few other diseases, an useful and effective remedy; yet we know that it is not possessed of such active powers over the animal body as mercury is; and, therefore, that when mismanaged, it will not be productive of as much mischief. Let us not impute to mercury more evils than those which really belong to it: let us draw a distinction between the judicious use of this powerful medicine, and the mal-administration of it. When we recollect how very universal the venereal disease is, how very numerous its symptoms in every one of the stages of this disease, how strangely these symptoms are modified by the habit of the patient, or by accidental circumstances—ex. gr. by inflammation; when we call to mind the astonishing powers of this medicine over the animal economy; and when, in addition to all these considerations, we reflect upon the immense number of injudicious, ignorant, uneducated persons, who fearlessly and constantly venture to undertake the treatment of syphilis by mercury, surely the wonder should be that it has not done infinitely more mischief in such hands." 322.

As we wish to disseminate as correct notions as possible on the subject of lues venerea, we shall pursue, as methodically as circumstances will permit, the plan at which we have hinted, and, as well as we are able, exhibit the course and characters of the disease—the effects of mercury, and the principles which should regulate our administration of it—and, finally, we shall touch on those debateable cases which are grouped under the generic designation of cachexia. It would be out of the question, attempting to embrace so many, so difficult, and so important topics in one, or indeed in two articles, and we shall make them the subject of as many as they seem naturally to require, in successive numbers of this Journal.

### 1. Natural History of Syphilis.

We shall commence, adopting the phraseology of Dr. Colles, with the natural history of syphilis. He devotes to it the first chapter in his book.

It may readily be granted, that morbid, like healthy action, has its laws. The phenomena of other diseases communicable by inoculation would induce us, à priori, to suppose that syphilis, like them, must naturally have a tendency to follow a definite course. But this has not, in the case of syphilis, been ascertained with the same precision as in that of small-pox, measles, or scarlet fever. The reasons must be plain. Those complaints run a rapid course, and when slight require no medical treatment, when severe are little modified by it. The opportunities for watching them, from their commencement to their termination, are consequently numerous. But syphilis pursues a very slow course, and it is much altered by medicine. From the former circumstance, patients are lost sight of, or they become disgusted if they make no progress, and obtain other advice—from the latter circumstance, mercury is either voluntarily or compulsorily given, and the opportunity of watching the progress of the disease is lost. These considerations not only explain the imperfect acquaintance we have yet attained with respect to the characters, course, and consequences of syphilis, but they explain also the difficulties that still attend its study.

Dr. Colles remarks, that :—

"We meet with nothing like a scientific description of the venereal disease in any of the earlier authors; in their writings we find only a mere enumeration of various local symptoms, without any arrangement or reference to their order of succession, and without any account of the course which any one symptom pursues through its different stages. Strange as it must appear, yet it is most true, and has proved most unfortunate for our science, that, prior to Mr. Hunter's treatise on the venereal disease, the profession possessed no systematic account, no accurate description either of its primary symptoms, or of the period or the order in which its secondary effects usually occur. This invaluable treatise poured a flood of light, not only on the natural history of this disease, but also on its pathology and treatment. It was Hunter who first demonstrated with clearness and precision that secondary symptoms succeed the primary at an interval of six or seven weeks, and that they are generally preceded by an eruptive fever, which is ordinarily of an inflammatory type: he also described the third order of symptoms; and all this he has done with an accuracy and fidelity which the subsequent experience of the profession has amply confirmed.

It is much to be lamented, that Mr. Hunter did not prosecute his enquiries still further; that he did not, for example, trace the full progress of secondary symptoms, or mark their natural tendency to amend, and then again to relapse, each unfavourable change being preceded by constitutional disturbance, and that he has omitted to notice the probable period of time which these symptoms occupy from their origin to their acmé, and thence to their decline. Whether these omissions proceeded from want of time, or from his having adopted the opinion that the venereal disease 'had no tendency to cure itself, or that the constitution was unequal to the cure of this disease,' is now of little moment. But does it not appear strange, that subsequent writers have not made some effort to supply these deficiencies?" 4.

We cannot say that we participate with Dr. Colles in his admiration of Mr. Hunter's Treatise on the Venereal Disease. That treatise exhibits in the most striking manner the vices of his mind—an insatiable thirst of speculation, and the contradiction or evasion of very obvious facts when they interfere with his views. Bad as Mr. Hunter's style of reasoning is on too many occasions, it is peculiarly so in the Treatise on the Venereal. We grant that it should be compared with what had gone before it, and that, when put into such a scale, it will be found to possess very weighty merits. But, taking it as a whole, we repeat that it is one of the worst of the treatises of Hunter. Take, for example, the following passage, in which Mr. Hunter denies that a child can be infected by the milk of an infected nurse.

"The breath and sweat are supposed to carry along with them contagion, the milk of the breast is supposed to be capable of containing venereal poison, and of affecting the child who sucks it; but there are several reasons which overturn these opinions. First, we find that no secretion is affected by this poison, excepting where the secreting organs have been previously affected by venereal inflammation or irritation, or its specific mode of action. Again, if they were contaminated so as to produce matter similar to that of an ulcer in the throat, such matter would not be poisonous, nor possess a power of communicating the disease, as will be explained more fully hereafter." 384.

The point at issue is either a fact or it is not. If it is a fact, theoretical considerations will not upset it—if it is not a fact, they are scarcely requisite. What does Mr. Hunter do? He does not look at the question as one of fact at all, but he objects to it an unintelligible and absurd argument touching the secretions—an argument to which this answer may at once be given that we know too little of the matter, to admit of any petitio principii, or to receive any analogical consideration as conclusive.

As a pendent to this sample of Hunter's mode of reasoning, we may cite another passage a little farther on:—

"It is also supposed that a fœtus in the womb of a pocky mother may be infected by her. This I should doubt very much, both from what may be observed of the secretions, and from finding that even the matter from such constitutional inflammation is not capable of communicating the disease. However, one can conceive the bare possibility of a child being affected in the womb of a pocky mother, not indeed from the disease of the mother, but from a part of the same matter which contaminated the mother, and was absorbed by her; and whether irritating her solids to action or not, may possibly be conveyed to the child, pure as absorbed; and if so it may affect the child exactly in the same way it did or might have done the mother." 385.

Here, again, we see a question of fact decided by speculation, and either confirmed or opposed by the most vague analogy and most unconditional hypothesis. We have never been amongst the extravagant admirers of Mr. Hunter, and, however heterodox it may appear, we must say that we look upon his principal merits as those of an anatomist and physiologist. A man of little original education, and imbued with that contempt for learning which is usually characteristic of vulgar ignorance, his writings frequently display the substitution of terms for ideas, and of words for things, and afford a pregnant instance of the disadvantages under which a strong mind labours, when undisciplined by sound education.

We do not then lament, with Dr. Colles, that Mr. Hunter did not pursue the subject farther. Pathology had made so little advance, and Mr. Hunter's mind was so constituted, that we think no great good would have come of his labours. He would probably have plunged deeper and deeper into speculation, and the authority of his name would have rivetted many serious errors on the minds of the profession; for, dogmas consecrated by authority are more difficult of removal than ignorance.

We believe that it will be of little service to pursue Mr. Hunter through his hypotheses. What good he really effected has become public property, and is incorporated with prevalent doctrines—what errors he committed are either known to be such, or will be discarded with the advance of knowledge, and must be attacked as substantive evils. We dismiss, therefore, Mr. Hunter's work with this reflection, that although it should be studied by every well-informed surgeon, it is not adapted for those who are not practically conversant with the complaint of which it treats. It should rather enter into a course of reading on the history of the venereal, than form a part of the practical study of it.

Dr. Colles describes what he has actually ascertained of the progress of syphilis, when unchecked by the action of mercury. It does not amount to much, because, as we have already explained, the obstacles to our attaining precise information are almost insuperable.

"I have observed," he says, "that when the local symptoms have become fully established, which is probably in two or three weeks after they make their appearance, they then become stationary, and the constitution is relieved from the febrile disturbance. In this quiescent state the symptoms may remain for about three weeks, they will then show a strong disposition to amend, and sometimes they will proceed so far, as to impress the surgeon with a hope, and the patient with a firm conviction, that he is about to get perfectly well. But this illusion, having lasted for two or three weeks, is dispelled in general by a fresh attack of eruptive fever, and by an eruption or sore throat, although sometimes other symptoms, (iritis for example,) be added to those under which the patient had been previously suffering. How long the disease might continue in this condition of alternate improvement and deterioration, I cannot pretend to say, as I have not had an opportunity of witnessing in the same individual more than two, or at the utmost three, of those revolutions: from what I have seen of such cases I am led to say we may calculate upon each relapse as likely to recur every third month. These remarks apply to the secondary symptoms as observed for two, three, or four of the first revolutions.

At a later, and sometimes, though rarely, in an early stage of the disease, we find a somewhat different process ushering in a new attack; thus we sometimes observe that the patient, who during the four or five preceding weeks had been improving in flesh, colour, appetite and strength, now begins to exhibit a different aspect; his countenance alters considerably, it becomes sickly, his complexion assumes a waxen hue and he evidently loses flesh from day to day, and all this takes place while the patient himself is not at all aware of the change, and is still less suspicious as to its cause. In some time, however, the patient complains of night sweats, want of sleep, loss of strength and declining appetite, so that in the course of two or three weeks he is reduced to a state of great weakness and emaciation: this downward course proceeds steadily until a fresh order of symptoms appears and becomes established.

I must here remark that I have repeatedly observed these newly appearing symptoms to be, as it were, a repetition of those that preceded them: thus, for example, a patient may have had a papular eruption which remained stationary for two or three weeks, then began gradually to decline, so as to lead him to hope he was about to become totally free from it; but this eruption having faded to a certain extent, has then become stationary for two or three weeks, and the general health has improved; at the end of this time, however, the health again begins to suffer in the manner above described, nor does it cease to decline until this new train of symptoms has become for some time established. More commonly, however, this long-continued wasting is followed by some new symptoms, often by those of the third order, succeeding the repeated renewals of secondary symptoms. After an attack of this lengthened nature we seldom if ever observe a distinct eruptive or premonitory fever appear in this patient; and I may also add that the local syphilitic symptoms cease to present their characteristic signs as strongly marked as heretofore. Thus it would seem as if each eruptive fever, or rather each succeeding attack, brings the constitution into a weaker and weaker state. In the very advanced stages of the venereal disease we do not see those periodical changes; the constitution then appears unequal to any struggle, so that one continued and increasing state of debility, with slow fever and great emaciation, are conjoined to the local symptoms, while the latter also are but little disposed to undergo any change, except a slow and gradual deterioration: thus, I have sometimes, though rarely, seen a tubercular eruption, combined with pains of bones and joints, continuing during four or five years, and undergoing no material change, being at one time a little better, and at another a little worse." 10.

This description very nearly represents what we have ourselves observed. In the cases which we have witnessed, the following circumstances were more or less constantly present.

1. The primary sore has usually, after some weeks, healed, and has, in the majority of instances, left in its site an indurated and a reddish-coloured cicatrix. In some instances this cicatrix has shewn a disposition to be covered with a slight crust or scab, the consequence of superficial excoriation and secretion. In other instances, the cicatrix has re-ulcerated once or oftener and at uncertain intervals. In women, it is not uncommon for a succession of sores to form. These, if on the labia, are usually succeeded by indurated cicatrices—if deeper the induration is much less perceptible. We would observe that, so far as our experience has gone, it is the rule for the primary sore to heal in men, and not to heal in women.* The reason *may* be, that men usually apply dressings of some sort to their sores, and that women commonly do not. The heat and moisture and lodgment of discharge are greater too in the latter than in the former.

2. From three weeks to three or four months after the distinct development of the primary sore, pyrexia and constitutional disturbance have ushered in either ulcerations of the throat, or papular, tubercular, or scaly eruptions on the skin; not unfrequently both ulcerated throat and eruptions. The usual characters of the ulceration of the throat have been—the tonsils and palatine arches tumid and red, and the ulceration itself yellowish and rather deep. The scaly eruptions (psoriasis) appear to us to have been the most frequent.

3. Irregular attacks of eruption, generally preceded by some pyrexia, and accompanied by pallor, and obvious ill-health, have continued for a variable time. Other symptoms may occur about this period. The patient may be affected with iritis—or with secondary ulcerations of the rectum—or with condylomata—or with venereal inflammation of the testis. It is generally about this period, that mercury is perforce resorted to, and here, consequently, we find it difficult to pursue the effects of the simple venereal poison.

4. Affections of the fibrous and osseous tissues are the next in order of occurrence. Not that the patient must necessarily have gone through the stages of sore throat and eruption previously to nodes. We have at this moment a gentleman under our care, who has node on the forehead, and below the great trochanter of the left femur, who had a sore which was pronounced by Mr. Lawrence to be venereal, who was prescribed mercury, but did not take it, and who has had no intermediate symptom. But as a general rule, nodes are preceded by eruptions and by ulcerated throat.

It is usually in combination with disease of the periosteum and bones, or

* We are speaking of cases in which mercury has not been exhibited, and in which no active treatment has been pursued.

just prior to their appearance, or about the period at which they occur, that another order of affections of the skin and of the mucous membranes is developed. Phagedænic ulcerations invade each, giving rise to frightful ravages in the throat, mouth, nose, and external parts of generation, as well as to rupia and spreading ulcers on the skin. Yet not only may nodes be unattended with these, but these may be uncombined with nodes. Both depend on a similar state of constitution, or are attended with it—a state of constitution eminently bad. It is true that such symptoms as we have alluded to, are generally seen in persons who have taken mercury to excess. But we have seen two instances, at least, in which they occurred independently of the use of mercury at all. The patients were both females, and, singularly enough, neither was conscious of any primary infection. It was this which prevented the administration of mercury.

It is probable that these are not ordinary results of the non-mercurial treatment, for whilst we constantly see them where mercury has been abused, we very seldom witness them where it has never been administered. But it must be recollected, that long before such symptoms naturally occur, the nature of the complaint has usually been rendered evident, and mercury has been exhibited.

5. We have only seen one person die with venereal symptoms absolutely unopposed by mercury. That patient was a female, and one of those to whom we just alluded. She died of phthisis. As this became developed, the nodes under which she laboured assumed less and less importance, and ultimately were scarcely thought of.

But it would seem that Dr. Colles has witnessed more numerous examples of a fatal termination to the malady. He remarks that:—

"When the disease has arrived at that advanced stage in which the general health has been broken down, and the local symptoms are so much changed as scarcely to be recognized as venereal, it is still a matter of uncertainty how long the patient (if not relieved by art) is to drag on a miserable existence, or in what manner a termination is to be put to all his sufferings. Many of its unfortunate victims are destroyed by what may be considered a continuation of the disease; but by far the greater number appear to be carried off by other complaints to which we may presume they were naturally disposed, or to which they were rendered liable by the very weakened and reduced state of their general health.

Among the former we may mention an ulceration of the throat, which, creeping downwards, at length seizes upon the larynx, and causes a destruction of a greater or lesser portion of this organ. The lungs quickly suffer from this affection of the larynx, and the patient dies, seemingly worn out by difficult respiration, and by severe cough, with profuse expectoration and hectic fever.

Others again are destroyed by the repeated exfoliation of the bones, frequently of those of the cranium, and sometimes of the long bones. Moreover, there are others who, having been forced by severe nocturnal pains to have recourse to opium, cannot afterwards be prevailed on to relinquish this medicine, but are rather disposed to increase the quantity of each dose. Although now almost free from the pains which had driven them to adopt this medicine in the first instance, such persons, we remark, cannot be induced to rise until late in the day; they have no appetite for breakfast, but will probably take some highly seasoned food for dinner; in the early part of the day they are quite torpid, averse to every kind of exercise; in the afternoon they seem to revive, become more animated in the early part of the evening, and are unwilling to retire to rest until a very late hour. One or two years may pass on in this manner with little apparent change; at length they sensibly decline in flesh, a diarrhœa supervenes, which is sometimes attended with sickness of stomach and vomiting, and by this they are ultimately carried off. It is remarkable that from the time these patients give themselves up to the inordinate use of opium their symptoms exhibit but slight traces of a venereal character.

Many others are carried off by diseases which have no other connexion with the original venereal affection except that by it, or rather by the treatment adopted for its cure, their system has been rendered more susceptible to the attacks of disease in general. Perhaps the greater number of such sink under attacks on the chest, such as pleuritis or pneumonia, terminating in serous effusion. Many of those who are strongly predisposed to phthisis fall victims to that disease: not a few are found to have the liver enormously enlarged, extending across the abdomen, and even below the umbilicus; this is followed by slow emaciation, and after some months by ascites and anasarca. Dysentery also at times seems to destroy some of those who have very long suffered under Syphilis, and, as might be expected, ulceration of the intestines is discovered after death.

From this account then it is very evident that there is no certain period after the appearance of the primary disease at which death terminates such protracted cases of Syphilis. We may say that in general this event takes place between the second and fifth or sixth year." 13.

This is all which it is in Dr. Colles' power to communicate with respect to the natural history of syphilis. It must be owned that the amount of positive information is not great, and that much remains to be done before our acquaintance with the natural and uncomplicated consequences of the poison can be considered satisfactory. The great obstacle to our acquisition of such knowledge is the time which the disease takes to run its course, an obstacle so serious as to render direct experiments of comparatively little value. A person may be got to submit to inoculation with the venereal poison, and to suffer secondary symptoms to shew themselves; but no person can be procured who will voluntarily let the latter symptoms go on to the destruction of his health, and perhaps of his life, and no surgeon could be such a scoundrel as to make an experiment to which the patient is not a consenting party. Experiments then, so far as they have gone, have done no more than establish what was partially or wholly known before, the communicability of such or such a sore, and the occurrence of a few of the earlier secondary symptoms; nor do we look to such experiments as likely in any material degree to augment our stock of knowledge. The great means of doing that will be the close and unprejudiced study of the experiments which Nature herself conducts for us, and an accurate record of the observations which we make.

Before he concludes the chapter on the natural history of syphilis, Dr. Colles examines the question of the power of secondary symptoms to communicate the venereal disease. He cites two cases in which secondary venereal ulcers were the means of infecting other parties. It is well known that Mr. Hunter not only entertained a contrary opinion, but based much reasoning on the positive denial of the fact, another instance not only of Mr. Hunter having been wrong, but of his having decided a matter of fact by à priori and dogmatic speculation. The point may be considered established that secondary venereal ulcerations *are* capable of infecting those favourably circumstanced for the reception of the disease; but the laws of the transmission of disease in this manner cannot be considered as determined.

The next subject on which Dr. Colles treats is the administration of mercury, that is, the principles which should regulate its use. But it would be more natural to ascertain first the characters and properties of the venereal sore, before we apply to it such a remedy as mercury. The only Chapter in which primary sores are considered is the fourth, professedly devoted to chancre. As the work of Dr. Colles is avowedly desultory, we will not quarrel with his arrangement, but pursue our own plan, and proceed to the examination of his views on the primary sore.

### II. On Chancre.

We cannot compliment Dr. Colles on the possession, or, at all events on the display of much method in his descriptions. That logical mode of so arranging our ideas that others may with the greatest facility comprehend them, is not an art which he appears to exhibit. Yet it is an useful one notwithstanding. We will endeavour to throw together observations which are rather unconnected in the original.

1. "Although every surgeon must admit that Mr. Hunter's description of a chancre is correct, and drawn from nature, still I believe few will confine this term, or that of primary venereal sore, to those ulcers only which answer to his description. As the result of long, attentive, and anxious observation, I should say that primary venereal ulcers present an almost endless variety of character. I would define a primary venereal ulcer to be 'one which is remarkably slow in yielding to ordinary, mild, local treatment, but which is curable by mercury, and which, if not so cured, is likely to be followed in two or three months, by secondary symptoms, which again also are curable by mercury.' If then there be, as I affirm there is, an almost endless variety in chancres, how can we decide on the nature of primary ulcers, so as to pronounce some to be syphilitic, and others to be mere common sores, or simple excoriations? I reply that we are to be guided in our decision, by observing, first, that many of these suspicious ulcerations cannot be referred to any class of common ulcers, as they strikingly differ from them; and, secondly, by attending to the course which these take, when not interfered with by any stimulant or caustic application, and when treated only with some mild ointment or cold water. If under these circumstances we find that after eight or ten days such ulcers show no disposition to heal, and if, at the same time there be a total absence of any cause, such as defect in the general health, to account for this obstinate condition of the local disease, we may then pronounce them to be syphilitic. But I repeat that the local applications must have been of the mildest kind, for almost any primary venereal ulcer may be made to heal by the use of stimulating applications, applied even for so short a time as one or two days." 76.

It will be observed that Dr. Colles abandons Mr. Hunter's hypothetical attempt, to erect one sore, and that not *now* the most common one, into the true type and symbol of syphilis. In this, all judicious surgeons will agree with him, and the old notions connected with the Hunterian chancre are virtually exploded.

But we cannot agree with Dr. Colles that venereal sores present an almost endless variety. In their minor features no doubt they do so, but in their leading characters they are not too diversified, to refuse, under a moderate analysis, to be reduced to a few leading forms. If pressed for an enumeration of them we would name the following:—

1. The Common Venereal Sore.
2. The Indurated Sore.
3. The Phagedænic Sore.
4. The Multifarious Sore.
5. The Excoriation Sore.

It is probable that these are not essential varieties quoad the nature of the poison, but rather, accidental modes of action, determined by numerous circumstances. A common sore, for instance, may become indurated or it may assume the phagedænic character. But, still, for practical purposes, it is useful to give a substantive character to ulcers which present very marked features, and require very marked varieties of treatment. It is foreign to our purpose to go into this subject at present, but we may observe that a variety of considerations hang by it, and that no simple view of the phenomena of venereal sores will either explain them, or be consistent with the truth.

It is obvious that Dr. Colles' definition of venereal sores is objectionable in many points of view. This is so obvious that we shall not insist on it, particularly as we care little about such things as definitions. But his directions for distinguishing venereal sores are of more practical consequence. The great criterion is their indisposition to heal. But that is a criterion which requires time, valuable time both to the patient and the surgeon. It is very well to wait for a week or a fortnight in a hospital, but private patients cannot or will not submit to such an ordeal, and the surgeon who should wish them generally to submit to it would probably find them evince a marked preference for some more decided rival. The fact is that the only mode of distinguishing venereal sores is first to become acquainted with their leading characters. Such knowledge can neither be acquired nor can it be conveyed by words. It is useless to attempt either. All that can be done by description is this—to classify venereal sores in the most natural manner—to describe as accurately as possible their respective characters—and finally to give some general hints which may assist in forming a diagnosis that essentially rests on a precise and a practical acquaintance with the disease. It is astonishing how quickly a surgeon who has that practical knowledge may pronounce, and accurately too, on the character of the vast majority of sores presented to him, at a very early stage of their existence.

We agree to a great extent with Dr. Colles in the following remarks:—

"It often happens that young men affected with primary sores, will first treat them for one or two days with stimulant washes, and then apply to a surgeon; he directs the mildest applications, and finding the ulcers to heal under this treatment without any hardness remaining, concludes that they were not venereal; the appearance of secondary symptoms, however, in a few weeks afterwards, proves the error of that opinion. I have known a few such cases, in which the surgeon has felt so confident in this opinion, that he has allowed the patient to marry in a short time after the healing of the sores. I need not attempt to describe the state of his feelings when called on to treat the wife for primary venereal sores!! I am therefore anxious to impress this as a very important rule, not to pronounce any ulcer on the genitals, as of an inocuous nature which may have healed under the mildest applications, if at any time, even during the short space of one or two days, it have been treated with stimulant applications." 76.

The early use of caustic has been recommended by some surgeons, especially by Mr. Wallace. We think it injudicious practice upon two accounts:—first, it interferes with the natural progress of the sore, and consequently with one important means of regulating our exhibition of mercury; secondly, it is apt to occasion much irritation, and the undesirable consequences which may ensue from it. Caustic at a later period is open neither to the one objection nor the other.

2. " I deem it right," says Dr. Colles, " here to notice an appearance in chancres healed by local means only, which we occasionally meet with in cases where mercury had been injudiciously employed,—an appearance which I have never, except in one instance, seen. The appearance I allude to is a remarkably hard lump, as large as a filbert, situated on the prepuce in the site of the original chancre, the skin which covers it is of a peculiar purple colour. In one instance, that of a young gentleman, whose chancre had been healed for three or four weeks, this appearance was attended with most alarming symptoms, viz. extreme emaciation, thirst, loss of appetite, and profuse night sweats. The suddenness with which he had been reduced to this state, from a tolerably full habit of body, and from apparently excellent health, excited in my mind great apprehensions for his safety, especially as some of his family had died of hæmoptysis succeeded by phthisis. I never saw any case in which the venereal virus acted in a manner so like to what we might fancy should be the effect of a slow poison on the system. Yet by means of a mercurial course gradually raised from very small doses, and continued for a longer period than usual, the hardness and discoloration were removed, and he was soon restored to good health, which he still enjoys. This case occurred many years since; at the time when the non-mercurial treatment was first introduced into Dublin. In all the other instances of this large purple tumor, where it has formed after the healing of a chancre by local means only, I have seen secondary symptoms follow in the same time, and pursue much the same course, as when these have succeeded to chancres which have been only imperfectly cured by the injudicious administration of mercury." 92.

This is an allusion, but it cannot be looked on as much more, to a subject of much practical importance, the condition of the cicatrix of a venereal sore. We are too much crippled for space at present to consider it at large, and we merely quote this passage, which, so far as it goes, deserves attention.

Dr. Colles alludes to those varieties of venereal sore, which depend on the peculiar structure of the part where they are situated. No doubt the nature of the part exerts an influence on the characters of the sore, and this is one element in the causes of the diversity of forms which sores assume. But we are not sure that it is so important an element as Dr. Colles' remarks would lead the reader to suppose. We will allow Dr. Colles to speak for himself.

3. It is scarcely necessary, he observes, to say that the quantity of hardness is much less in chancres situated on the glans than in those on the prepuce; but still a degree of hardness sufficient to guide us in the treatment attends even the former.

No doubt some induration attends the venereal sore on the glans. But Dr. Colles takes no notice of several peculiarities attending such a sore. In the first place, granulations are generally tardy, and such a sore not unfrequently assumes the phagedænic character. In the second place, such a sore scarcely ever fungates, the absence of loose cellular tissue beneath the compact chorion, interfering, we presume, with the ready formation of loose granulations. In the third place, the cicatrix is generally depressed, rather than elevated, and the loss of substance is seldom properly repaired. Even after the lapse of years a hollow is seen to mark where the sore was.

"There is a form of venereal chancre on the edge of the prepuce, of which I do not recollect ever to have read any description. The account we receive from the patient is, that he thinks he tore himself in the act of connexion; we see that the lacerated part shows no disposition to heal, but is not painful or swollen, and appears in the form of a fissure. When the prepuce is partly retracted, this fissure appears as if it had been divided by a long incision; the edges are hard and deep, not swollen or inflamed, and yielding scarcely any discharge; it is not at all painful, unless when an attempt be made to retract the prepuce. This is certainly a primary venereal ulcer, its peculiar characters may probably depend on the infection being ingrafted on the torn surface; it requires the use of mercury to the same extent as does the circular Hunterian chancre: nor does it need any peculiar local treatment." 93.

This form of sore is very common in the London Lock Hospital. It not unfrequently coincides with gonorrhœa—almost always occurs in persons who have a long prepuce and a tendency to natural phymosis—but, so far as we have seen, is not necessarily preceded by excoriation. It is, undoubtedly, a form of venereal ulceration, and we have seen it followed by ulcerated throat, and by papular, tubercular, and scaly eruptions. Phymosis is not uncommonly occasioned by it.

"We sometimes see the edge of the prepuce beset with five or six circular ulcers; these, if left to themselves, will first granulate, then become fungous, and finally will heal spontaneously. The form as well as the slow and indolent character of these ulcers might dispose us to conceive that they were syphilitic; the appearance, however, of several of these at once, along the margin of the prepuce, and their being destitute of any hardness, will serve as a criterion whereby we may make the distinction. These ulcers may always be cured by suitable local treatment alone; I have never seen them take an unfavourable turn. Sometimes there may also be present in conjunction with these, others of a true venereal character, and for which mercury must be administered; we shall then have an opportunity of remarking that this medicine exerts no influence on the ulcers at the extremity of the prepuce. Sometimes this circle of ulcers is accompanied by a single one of the same character far within the prepuce." 94.

We feel some hesitation in coinciding with Dr. Colles in his opinions on this form of ulceration. The multifarious, fungating ulcers on the prepuce, are, so far as our experience goes, venereal. Dr. Colles gives no marks by which we may suppose that they are not so, save the fallacious one, that they will *finally* heal spontaneously. They will do so finally, no doubt, and so will most of the true venereal ulcers. But we are sure that in the majority of cases they will take a very long time to heal, and that if allowed to do so spontaneously, secondary symptoms will in certain number follow. The fact is, that this form of venereal ulceration is rather a peculiar one, and we have made it the subject of a distinct essay, in which we have described its characters and progress, pointed out what seems to us the most successful mode of treatment, and alluded to some of its natural consequences.

Dr. Colles remarks that the non-venereal are sometimes mixed with true venereal ulcers, but he gives us no criteria by which we may distinguish them, and it is singular that two forms of sore, one venereal and the other not so, should be running abreast, in so friendly a manner. One would imagine that the discharge of the venereal sore would at once contaminate the non-venereal one. We fear that there is on this, as well as on other occasions, rather too much looseness in Dr. Colles' descriptions.

6. "When a chancre is seated close to either side of the frænum, we find it invariably happens that the ulceration passes through this process of integument, at first making an opening through it, and in a few instances the ulcer has healed, leaving this hole; but in the majority of cases the ulceration goes on until it has divided the frænum entirely, then each side of the ulcer heals separately, the part on the glans showing a tendency to advance slowly, making a superficial groove in the anterior part of the glans. The only direction to attend to in the management of these chancres, is to cut through the frænum as soon as the ulcer has once perforated it; for we shall generally find when this has not been submitted to, that the sore will not assume the granulating state, until the ulceration has completely divided the frænum. The posterior part of the ulcer having generally the larger part of the frænum, will require repeated touches of caustic to prevent it from healing with a large knob, while the anterior part will be benefited by the black wash or some other gentle stimulant. Sometimes the frænum (having been divided by the surgeon or by ulceration) presents a furrow, showing that the part is composed of two layers of cuticle; this may be a little more tedious in healing." 94.

We cannot look on this as an adequate account of the perforating ulcer of the frænum, a very marked variety of the common venereal sore. The ulcer in question runs a very peculiar course, always, or almost always, perforates the frænum, and destroys the portion of it which is rendered free by the perforation, and not very unfrequently assumes the phagedænic character. We have seen in private practice, during these last six months, five cases of it, and of those, two had taken on the phagedænic aspect, and creeping or "herpetic" character. It would occupy us far too long, for our space is now exhausted, to go into the history and treatment of this sore. Suffice it to say that it requires the cautious and the long-continued use of mercury. In one instance in which mercury was imperfectly administered, we saw suppurating bubo in each groin, ulcerated (yellow) tonsils, and tubercular eruption follow. The case was that of a medical student.

7. "A chancre sometimes, though rarely, is seated at the very orifice of the urethra; in this situation it is always slow in going through the different stages preparatory to healing, and it too often assumes an eating disposition, passing all round the orifice; there is one very serious ill consequence, which always results from this, namely, that in some time, perhaps in one or two years, the patient will suffer severely from symptoms of stricture. In treating such a case, we should use every effort to prevent the extension of the ulceration to the entire circle of the orifice, for unless it entirely encircle the orifice, contraction will not follow; this can with certainty be accomplished by touching the ulcer as soon as it begins to extend, with the colourless muriate of antimony, or with nitric acid: these applications are no doubt severe, but the evil they avert is one of great magnitude; for I will venture to assert that of all forms of stricture, this is one most apt to recur; indeed it does not in any instance admit of a cure by the ordinary treatment of strictures.

"I am happy to say that I have lately discovered a mode of treating this stricture which has proved eminently successful in the few cases in which I adopted it. This plan of treatment consists in this simple operation.—Having detached the skin from the end of the urethra, to which it is generally intimately adherent, I divide the urethra below, to the length of more than half an inch. I raise the mucous membrane from each lip of the incision, then cut away a portion of the bared corpus spongiosum, to such an extent as will allow the raised mucous membrane to cover the cut edge. I stitch down this membrane upon the corpus spongiosum; and thus having covered each lip of the wound by mucous membrane, I have effectually guarded against the possibility of re-union of the lips of the wound, or subsequent contraction of the opening. The opening of the urethra thus produced, is of course of a size larger than natural."

This is a valuable suggestion, and we shall adopt it in the first case, of an appropriate description, which presents itself. As Dr. Colles observes, the stricture produced by ulceration of the orifice of the urethra is of a very troublesome description, but it does not always succeed such ulceration. We have at this time a gentleman under our care, in whom such ulceration has healed without any tendency to constriction, and in another gentleman, labouring under stricture of the membranous part of the urethra, a venereal ulcer at its orifice has enlarged *that* materially.

8. "Of late years, notwithstanding what Mr. Hunter asserts to the contrary, I am confident that I have seen cases of chancre seated altogether within the urethra; such cases have been frequently mistaken for mild gonorrhœa, and have for weeks together been treated as such: in some of these cases the surgeon has not been apprized of his mistake until the ulceration has actually laid open the fore-part of the urethra, or has extended forwards to its orifice, so as to become visible. A very little attention will enable us to discover the real nature of this case, even in its earliest stages; for we can externally feel a hardness in that spot of the urethra where the chancre is seated, and the symptoms of gonorrhœa are so slight, that this alone should excite our suspicion, and induce us to search for a chancre in the urethra." 96.

Nothing can be more true than the preceding observations, and nothing can be more corroborative of what we have asserted respecting Mr. Hunter's work on the venereal. It did not agree with his speculative views to admit of a venereal ulcer of the urethral mucous membrane, and consequently he denied its existence; in other words, he decided, as he often does, a question of fact not by reference to facts, but by theory—the most illogical and unphilosophical method of argument possible. It hardly, we imagine, requires additional evidence to prove that Mr. Hunter's mind, much as it has been lauded, was not imbued with the spirit of inductive reasoning, for such a mental vice as we have mentioned, is incompatible with its very principles.

We have ourselves, within the last four years, seen three distinct cases of venereal ulcer within the urethra. Of these, two were in the Lock Hospital, and were out-patients of our own, and one was a private patient. In the first case, we mistook the disease for gonorrhœa, until the occurrence of ulcerated tonsils, and syphilitic psoriasis, drew our attention more particularly to it, and led us to make a closer examination of the urethra. In the other two cases, the same mistake had been made by other surgeons—in the private patient by a very eminent one. Those two patients had secondary symptoms too; one laboured under stains and desquamating papulæ, the other under pityriasis with psoriasis, and ulceration of the tonsils.

Here we pause. In our next number we shall pursue the subject, and we hope to offer some observations which may be of service to such of our readers as have not enjoyed sufficient opportunities of studying this complicated malady.

---

### RECENT WORKS ON THE PRACTICE OF MEDICINE.

